# Ruminal pH Dynamics and Milk Production Response to Concentrate Supplementation in Pasture-Based Dairy Cows

**DOI:** 10.3390/ani16121771

**Published:** 2026-06-08

**Authors:** Romina Rodríguez-Pereira, Natalie L. Urrutia, Emilio M. Ungerfeld, Isadora A. Muñoz, Camila Muñoz

**Affiliations:** 1 Escuela de Medicina Veterinaria, Facultad de Recursos Naturales y Medicina Veterinaria, Universidad Santo Tomás, Talca 3460000, Región del Maule, Chile; rrodriguez22@santotomas.cl; 2 Centro Regional de Investigación Remehue, Instituto de Investigaciones Agropecuarias, Osorno 5290000, Región de Los Lagos, Chile; natalie.urrutia@inia.cl (N.L.U.);; 3 Centro Regional de Investigación Carillanca, Instituto de Investigaciones Agropecuarias, Temuco 4880000, Región de La Araucanía, Chile; emilio.ungerfeld@inia.cl

**Keywords:** grazing, SARA, ruminal acidosis, enteric methane, pasture, cows, milk, rumen, pH, concentrate

## Abstract

The risk of subacute ruminal acidosis (SARA) in grazing dairy cows remains uncertain, particularly when concentrates are supplemented. This study evaluated the effects of concentrate supplementation on the evolution of diurnal ruminal pH and its relationship with production, nutrient utilization, digestive indicators, and health status. Eight ruminal-fistulated multiparous Holstein–Friesian cows were assigned to a 2-period crossover design comparing a pasture-only diet (PO) and the same pasture supplemented with 6 kg/d of grain-based concentrate (PC). Each 28 d period included 21 d of adaptation and 7 d of measurements. Cows were fed freshly cut perennial ryegrass. Ruminal pH was recorded at 0, 2, 4, 6, 8, 10, 12, and 18 h relative to feeding. Concentrate supplementation slightly decreased minimum ruminal pH (5.97 vs. 6.15) but remained above the SARA threshold. Total volatile fatty acids (VFAs) increased, and acetate molar percentage decreased with supplementation. Total dry matter intake increased by 3.5 kg/d, increasing milk yield by 3.85 kg/d, and improving milk protein concentration, while milk fat and fatty acid profile, ruminal ammonium, and clinical indicators were unaffected. Ruminal pH was higher and VFA concentrations lower late in the season. Overall, concentrate supplementation was associated with improved productivity without compromising ruminal stability under the conditions of this study; however, responses were influenced by seasonal variation in pasture characteristics.

## Simple Summary

Dairy cows grazing fresh pasture are often given grain supplements to balance nutrition and increase milk production. However, farmers and veterinarians are concerned that these supplements could disturb the rumen by triggering subacute ruminal acidosis, a digestive disorder that is difficult to detect but can reduce welfare and performance. This study evaluated whether supplementing concentrate to a pasture-based diet affected ruminal acidity, digestion, milk production, and health indicators of grazing dairy cows. Eight cows were studied under two feeding conditions: pasture only or pasture plus concentrate feed. Feeding concentrate slightly increased ruminal acidity after meals, but levels remained within a normal and safe range. In addition, cows ate more feed and produced more milk, particularly milk protein, without negative effects on milk fat or animal health. Seasonal pasture changes had a larger effect on ruminal conditions than supplementation itself. Metabolic indicators measured in manure and blood, and behavior assessments confirmed that cows maintained normal physiological status. Overall, the results suggest that concentrate supplementation may support productivity under pasture-based conditions without increasing the risk of digestive disorders, although responses were influenced by seasonal variation in pasture quality.

## 1. Introduction

Pasture-based dairy production systems are widely recognized for their capacity to convert human-inedible forage resources into high-quality milk in an economically and environmentally efficient manner, particularly in temperate regions, where high quality pastures support low-input dairy systems [1]. In these environments, dairy cows can graze fresh forage during most of their lactation, with pasture often representing more than 80% of dietary dry matter intake. However, concentrate supplementation is routinely used to sustain milk production and meet the energy requirements of high-producing cows [2,3]. When concentrates are combined with highly digestible temperate pastures, ruminal fermentation patterns may be altered, potentially challenging ruminal stability. Under these conditions, maintaining an optimal ruminal environment is critical for sustaining intake, nutrient utilization, and milk synthesis.

Rumen microorganisms ferment dietary carbohydrates into volatile fatty acids (VFAs), which supply most of the metabolizable energy to the host animal, while dietary nitrogen is converted into microbial protein. Because VFAs are precursors of milk fat synthesis, alterations in ruminal fermentation may influence milk fatty acid composition. Adequate ruminal function depends on the balance between fermentable substrates, effective fiber supply, and buffering capacity, with ruminal pH acting as a central integrative indicator of this balance [4,5].

When this balance is disrupted, either due to insufficient physically effective fiber or excessive intake of rapidly fermentable carbohydrates, ruminal pH may decline, increasing the risk of subacute ruminal acidosis (SARA). SARA is characterized by recurrent episodes of moderately depressed ruminal pH, typically below 5.8, although this threshold may vary depending on the criteria used and measurement methodology [6,7]. Typically associated with altered intake patterns, reduced fiber digestibility and milk fat synthesis, and negative health outcomes [7,8]. These effects are often subclinical and multifactorial, manifesting as subtle changes in performance and animal health rather than overt clinical signs. 

Historically, grazing dairy cows were considered at low risk of ruminal acidosis due to the high fiber content of fresh forage [9]. However, accumulating evidence suggests that cows grazing highly digestible temperate pastures may experience daily episodes of low ruminal pH comparable to those observed in total mixed rations systems [6,10,11]. Mean ruminal pH values below 5.8 and an increased prevalence of SARA risk have been reported under grazing conditions [10,11,12], suggesting that pasture-based systems may not always prevent ruminal acidosis, particularly when combined with concentrate supplementation.

Concentrate supplementation is therefore a key nutritional intervention in pasture-based dairy systems, with the potential to enhance energy intake but also to modify ruminal fermentation when combined with highly fermentable pasture [13,14]. Changes in ruminal pH may influence fermentation patterns, nutrient utilization, milk composition, and animal health [6,8]. Complementary indicators such as fecal pH, fecal particle size, and systemic acid–base variables may also provide additional insight into digestive responses, while metabolic indicators such as plasma glucose and non-esterified fatty acids (NEFAs) reflect the energetic status of dairy cows. In addition, dietary effects on ruminal fermentation may influence enteric methane emissions [15,16].

Accordingly, a comprehensive evaluation of ruminal pH dynamics along with animal performance and indicators of digestive function and health, is required to better characterize the effects of concentrate supplementation in fresh forage-based diets. In this context, cows received 6 kg/d of concentrate, a level commonly used in pasture-based dairy systems, although its relative magnitude may vary depending on grazing conditions.

The objective of this study was to evaluate the effect of concentrate supplementation on the daily ruminal pH pattern of dairy cows fed fresh forage-based diets, within the broader context of production performance, nutrient utilization, and health-related responses. We hypothesized that concentrate supplementation would reduce ruminal pH and increase the risk of subacute ruminal acidosis (SARA) in dairy cows fed fresh forage-based diets.

## 2. Materials and Methods

The experiment was carried out at the Instituto de Investigaciones Agropecuarias, INIA Remehue, located in Osorno (40°31′ S; 73°03′ W), Chile, during 56 days in the spring (October–December) of 2021.

All animal procedures were performed in accordance with the requirements of the Chilean Law 20,380 on Animal Protection and with the approval of the INIA Ethics Committee (Approval No 05/2021).

### 2.1. Animals, Experimental Design, and Treatments

Eight multiparous lactating, spring-calving Holstein–Friesian dairy cows fitted with rumen cannulas were used in a two-period crossover design with two dietary treatments. Each experimental period lasted 28 d, comprising 21 d of dietary adaptation followed by a 7 d measurement phase. Period 1 was conducted from 13 October to 9 November, and Period 2 from 10 November to 6 December. The 21-day adaptation phase within each period was considered sufficient to minimize potential carryover effects between treatments; therefore, a separate washout period was not included as extending the time between measurement periods would have accentuated seasonal variation in pasture quality and introduced additional confounding effects.

At the beginning of the trial, cows were blocked according to milk yield (26.6 ± 5.2 kg/d), days in milk (46.5 ± 15), and body mass (523 ± 68.4 kg), and randomly assigned to 1 of the 2 treatments: pasture only (PO), with cut and carry fresh pasture offered *ad libitum* plus a mineral supplement; and pasture plus concentrate (PC), with cut and carry fresh pasture offered *ad libitum* supplemented with 6 kg/d (as fed basis) of a commercial grain-based concentrate and a mineral supplement. The ingredients and chemical composition of the commercial concentrate are presented in Table 1. The mineral supplement (Nutrialmix; Nutrial S.A., Osorno, Chile) supplemented to all cows provided per kg of product: Ca 180 g, P 50 g, Mg 60 g, Na 12 g, selenium-enriched yeast 1 g, Cu 1200 mg, Mn 1500 mg, Zn 2700 mg, I 125 mg, Co 20 mg, and Se 30 mg. The mineral supplement was administered through the rumen cannula in PO cows and mixed with the concentrate in PC cows.

In the first 21 d of each period (adaptation period), the cows grazed fresh spring pasture, in two groups separated by treatment. Each paddock (3.3 ha) was subdivided into sub-paddocks for rotational grazing and cows strip-grazed using electric fencing. Pasture swards consisted of perennial ryegrass (*Lolium perenne*; 83%), white clover (*Trifolium repens*; 9%), other grasses and weeds (7%) and dead material (1%). The daily herbage allowance target was 20 and 14 kg of dry matter (DM) measured above 3 cm per cow/day for the PO and PC treatments, respectively. For both treatments, pre- and post-grazing herbage mass were measured with a rising plate meter (Farmworks, Feilding, New Zealand).

Cows were milked twice daily (06:30 and 16:00 h) and after the morning milking, they accessed a new pasture strip. Concentrate for PC cows was individually offered in 2 equal portions at each milking. There were no concentrate refusals observed during the study.

During the 7 d measurement phase of each period, cows were individually housed in a digestion–metabolism unit and fed freshly cut pasture harvested from their respective paddock (cut and carry) to mimic grazing conditions while allowing accurate individual intake measurements. Pasture was cut at approximately 7–10 cm above ground level using a motor scythe equipped with a sickle bar blade, collected as swaths, and offered directly to the cows as fresh forage (cut-and-carry method) without further handling. The pasture was offered to cows *ad libitum* once daily in the morning based on their intake from the previous day, allowing for 10% feed refusals (DM basis). Fresh water was available *ad libitum*.

### 2.2. Pasture Measurements, Fecal and Urine Collection and Digestibility Determination

During the 21 d adaptation pasture, DM was determined twice weekly to adjust fresh herbage allowance. During the measurement weeks, individual daily intake was recorded, and representative samples of the diets offered and refused were collected daily for 6 consecutive days. Samples were composited by day and treatment for subsequent chemical analyses. To assess digestibility, total fecal collection was conducted over 5 consecutive days. Urine was collected simultaneously as part of the total collection system inherent to the metabolism unit and acidified to preserve urinary nitrogen for subsequent analyses; nitrogen balance data are not reported in the present manuscript, and digestibility calculations were based exclusively on nutrient intake and fecal output.

Feces were collected using stainless-steel trays (100 × 120 × 20 cm) lined with plastic sheets and positioned directly behind the animals to prevent contamination and ensure complete recovery. Urine was collected in 25 L plastic containers connected via a flexible hose and funnel system attached with Velcro straps to adhesive patches placed around the vulva and rump, ensuring continuous flow [17]. To preserve urinary nitrogen during storage, urine was acidified daily with approximately 150 mL of 35% (*v*/*v*) sulfuric acid per 25 L container (volume adjusted according to daily output) to maintain pH ≤ 3.0, following Muñoz et al. [17] and AOAC recommendations [18].

Daily fecal and urinary outputs were subsampled at 5% of total fecal excretion (by weight) and urine output (by volume). Feces and urine were composited by animal across the 5-day collection period and stored at −20 °C until analysis. Composited samples of pasture offered, concentrate, refusals and feces were oven-dried at 60 °C for 48 h, ground to pass through a 1 mm screen (Wiley mill, 158 Arthur H. Thomas, Philadelphia, PA, USA), and analyzed for DM, crude protein (CP), and ash according to [18] (methods 934.01; 2001.11; 923.03; respectively). Amylase-treated neutral detergent fiber (aNDF) was determined following [18] (method 2002.04). In vitro digestibility was assessed using the technique of [19]. Nutrient intake was estimated as the difference between the amounts of nutrients offered and refused. Apparent in vivo digestibility coefficients of DM, organic matter (OM), nitrogen, and aNDF were calculated as the proportion of ingested nutrient not recovered in feces. The substitution rate was calculated as the difference in pasture DM intake between PO and PC divided by concentrate DM intake in PC [13]. 

### 2.3. Milk Measurements

Cows were milked twice daily, and individual milk yield was recorded automatically (DeLaval Alpro MM15; DeLaval International, Tumba, Sweden) during the adaptation periods. During measurement days, milk was collected with a mobile milking unit fitted with Waikato samplers and individual milk yields were weighed and recorded manually. Individual milk samples were collected on 6 consecutive milkings (AM and PM) of each experimental period. Samples from two consecutive milkings were combined for each cow in proportion to milk yield to obtain a representative daily composite. Milk samples were preserved with bronopol and stored at 4 °C until analysis (fat, protein, and urea) using infrared spectroscopy (Milkoscan FT6000, Foss Electric, Hillerød, Denmark). In addition, milk samples were analyzed through Near-Infrared Reflectance Spectroscopy (NIRS) for fatty acid composition according to Lobos-Ortega et al. [20] using NIR spectroscopy (MPA-FT NIR, Bruker Optik GmbH, Ettlingen, Germany). Milk yield was adjusted for energy content to calculate energy-corrected milk (ECM) using the equation proposed by [21].

### 2.4. Rumen Samples and Measurements

In order to establish the daily ruminal pH pattern, on day 3 of each measurement week, rumen samples were collected manually through the rumen cannula from 5 rumen sites (cranial ventral, caudal ventral, central, caudal dorsal and cranial dorsal) at 0, 2, 4, 6, 8, 10, 12, and 18 h after feeding and mixed and strained through 4 layers of cheesecloth for immediate measurement of ruminal pH with a portable pH meter (model exStik^®^ PH100, Extech, Nashua, NH, USA). In addition, samples of rumen fluid were preserved frozen at −20 °C for the determination of VFA concentration by gas chromatography. 

One-milliliter rumen fluid aliquots were transferred to Eppendorf tubes containing either 0.2 mL of 25% (*w*/*v*) meta-phosphoric acid (Merck, Darmstadt, Germany) for volatile fatty acids (VFAs) determination, or 0.2 mL of 1% (*v*/*v*) sulfuric acid for ammonium ion (NH_4_^+^) analysis and stored at −20 °C until analysis [22]. For VFA determination, samples were thawed, vortexed, and centrifuged at 16,000× *g* for 10 min, and the supernatant was filtered through 0.45 µm cellulose filters into 2 mL gas chromatograph (GC) vials. One microliter of filtrate was injected into a PerkinElmer Clarus 580 GC (PerkinElmer, Shelton, CT, USA) equipped with an Elite-FFAP capillary column and a flame ionization detector, using helium (1.5 mL/min) as the carrier gas. The oven program was set at 90 °C with a ramp of 12 °C/min up to 150 °C, held for 5 min. Ammonium concentration was determined colorimetrically.

### 2.5. Saliva, Urine and Fecal Samples

Saliva, urine, and fecal samples were collected for pH determination. On day 5 of the measurement week, samples of saliva and urine were collected twice (prior to feed allocation and 6 h after feeding). Saliva samples were obtained directly from the buccal cavity into sterile 50 mL plastic tubes. Urine samples for pH determination were collected separately from the total urine collection, either during spontaneous urination or after gentle manual stimulation of the vulva into sterile containers. Urinary pH was measured immediately after collection on spot samples and was therefore not affected by the acidification applied to total urine samples. Urinary pH was included as a complementary indicator of systemic acid–base status. Fecal samples (approximately 500 g) were collected through rectal palpation or natural defecation at 0, 6, 12, and 18 h after feeding for measurement of pH. After measuring pH, the remains of fecal samples (200 g) were used to determine fecal particle size by wet sieving. Each sample was wet sieved in duplicate using 3 stainless steel sieves (top, 4.75 mm; middle, 3.35 mm; bottom, 1.4 mm), a 12 L plastic bucket, and a gun spray nozzle [23]. The sieves containing the fecal particles were dried in a forced air oven at 60 °C for 48 h and then weighed. The dry feces, retained in each mesh size, were calculated as a percentage of the total fecal dry matter sieved. Fecal consistency was assessed using a 4-point dung score scale as described by [24].

### 2.6. Blood Samples

Coccygeal blood samples were collected twice daily on day 2 of each measurement week before and approximately 6 h after feeding. Blood was sampled into tubes containing heparin, EDTA, or sodium fluoride, depending on the subsequent analysis. Whole blood from heparinized tubes was immediately used for pH determination. Plasma obtained from EDTA-treated samples was analyzed for NEFA, whereas plasma from sodium fluoride tubes was used for glucose determination. Glucose concentration was measured using the PGO enzyme assay (procedure no. P7119; Sigma-Aldrich, St. Louis, MO, USA), and NEFA concentration was quantified colorimetrically using a commercial kit (Wako HR Series NEFA-HR; Wako Chemicals USA, Richmond, VA, USA), following the modified procedure described by [25].

### 2.7. Clinical Assessment

Body weight (BW) was recorded weekly using a calibrated electronic livestock scale, prior to the morning feeding, to monitor changes in liveweight across the experimental periods. Body condition score (BCS) was assessed concurrently by two trained evaluators using a 5-point scale with 0.25-point increments, where 1 = emaciated and 5 = obese, as described by [26]. Reticulorumen motility was evaluated twice during the sample collection week using auscultation. The frequency and intensity of reticulorumen contractions were recorded over a 2 min period in standing cows at rest.

Clinical examinations were conducted midmorning throughout the measurement week to assess animal health status. Parameters evaluated included, general demeanor, scored subjectively as alert, quiet, or depressed, rectal temperature, heart rate, and respiratory rate. All clinical measurements were performed by the same trained veterinarian to ensure consistency, and any abnormalities were recorded and managed in accordance with institutional animal welfare protocols.

### 2.8. Methane Emissions Measurements

Enteric methane emissions were quantified using the SF_6_ tracer gas technique [26] following the protocol described by [17]. Permeation tubes were placed in the rumen approximately 7 days before the collection period to stabilize release rates (3.83–4.22 mg/d). Gas samples were collected over five consecutive days using halter-mounted evacuated canisters, with background air sampled daily to account for ambient gas concentrations. Methane and SF_6_ concentrations were determined by GC (Perkin Elmer Clarus 600; Waltham, MA, USA), and daily methane emissions were calculated from the CH_4_:SF_6_ ratio, corrected for background concentrations.

### 2.9. Statistical Analysis

All data were analyzed using SAS OnDemand for Academics (version 3.81, SAS Institute Inc., Cary, NC, USA). Variables measured once per experimental period were analyzed using a mixed model including treatment and period as fixed effects and cow as a random effect.

Repeated-measures variables (e.g., ruminal and fecal pH and ruminal VFA concentrations measured at multiple sampling times) were analyzed using the MIXED procedure of SAS. The model included treatment, period, sampling time, and their interactions (treatment × time, and treatment × period) as fixed effects, and cow as a random effect. Repeated observations over time were specified using cow within period as the subject (i.e., cow within period) to account for the correlation among repeated observations within the same experimental unit. A first-order autoregressive covariance structure [AR(1)] was selected based on the lowest Akaike Information Criterion (AIC) and Bayesian Information Criterion (BIC). Denominator degrees of freedom were adjusted using the Kenward–Roger method. Least squares means were calculated for all fixed effects and compared using Tukey’s adjustment. When the treatment × time interaction was not significant, only main effects were reported. Statistical significance was declared at *p* < 0.05 and trends at 0.05 ≤ *p* < 0.10. Model residuals were evaluated for normality and homogeneity of variance using graphical diagnostics and the UNIVARIATE procedure of SAS.

## 3. Results

### 3.1. Chemical Composition and Nutrient Intake

The chemical composition of the pasture offered during the experimental periods is presented in Table 2. Pasture composition differed markedly between periods. In Period 1, pasture was characterized by a lower DM content (*p* < 0.001) and higher CP (*p* = 0.018), ash (*p* = 0.008), in vitro digestibility (*p* < 0.001), and metabolizable energy (*p* < 0.001) than Period 2. In contrast, NDF content did not differ between periods (*p* > 0.05).

### 3.2. Nutrient Intake and Digestibility

Concentrate supplementation increased total DM and organic matter intakes (*p* < 0.05). Crude protein intake showed a tendency to be greater in concentrate supplemented cows (*p* = 0.10), whereas NDF intake tended to be lower (*p* = 0.08). No significant differences were detected in apparent digestibilities of DM, OM, and CP between treatments (*p* > 0.05), and NDF digestibility was reduced in cows receiving PC (*p* = 0.004).

Period affected total DM, OM, and NDF intakes were greater in Period 2 (*p* < 0.05). NDF digestibility declined as the experiment progressed (*p* = 0.003), while digestibility of DM, OM, and CP remained unchanged (*p* > 0.05). No treatment × period interactions were detected for any variable (*p* ≥ 0.13; Table 3). The calculated substitution rate was 0.18 and 0.40 kg of pasture/kg of concentrate in Periods 1 and 2, respectively.

### 3.3. Ruminal pH and Fermentation Characteristics

**Figure 1:**
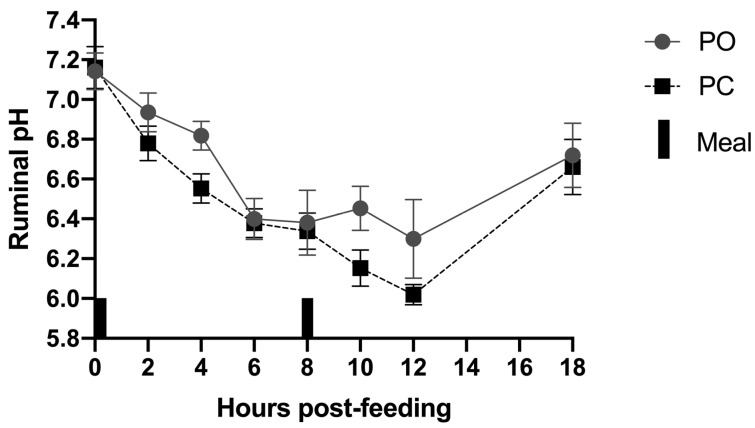
Diurnal pattern of ruminal pH in cows fed pasture only (PO) or pasture plus concentrate (PC), averaged across both experimental periods. Ruminal pH was measured at 0, 2, 4, 6, 8, 10, 12, and 18 h relative to morning feeding. Values represent least squares means ± SEM. No treatment × sampling time interaction was detected (*p* > 0.05).

A treatment × period interaction was detected for minimum ruminal pH (*p* = 0.027; Table 4), with cows receiving PC exhibiting lower minimum pH values than PO treatment during Period 2 but not Period 1. Concentrate supplementation reduced average ruminal pH (*p* = 0.032) without affecting maximum pH (*p* = 0.87; Table 4). The temporal variation in ruminal pH throughout the day, averaged across both experimental periods is shown in Figure 1.

Total VFA concentration increased in cows fed PC than PO (*p* = 0.005). This response was accompanied by a lower acetate molar percentage (*p* = 0.001) and greater butyrate molar percentage (*p* = 0.001), whereas propionate molar percentage and the acetate-to-propionate ratio were unaffected (*p* > 0.05). Valerate molar percentage increased with supplementation (*p* = 0.035), while isobutyrate was greater in PO (*p* = 0.039). Ruminal ammonium concentration was not influenced by treatment (*p* = 0.14).

Across periods, average and minimum ruminal pH were greater in Period 2 than in Period 1 (*p* ≤ 0.007). Total VFA concentration declined (*p* < 0.001), with higher propionate and lower acetate proportions observed in Period 1 (*p* < 0.05). Ruminal ammonium concentrations were also greater in Period 1 than in Period 2 (*p* < 0.0001).

### 3.4. Milk Production and Composition

Concentrate supplementation increased milk yield and energy-corrected milk (ECM) by approximately 3.85 kg/d in PC compared with PO (*p* < 0.01; Table 5). Protein and fat yields were also greater in PC (*p* ≤ 0.002). Milk protein concentration increased with supplementation (*p* = 0.01), whereas No significant differences in milk fatty acid composition were detected between treatments (*p* = 0.89), resulting in a lower fat-to-protein ratio in PC (*p* = 0.041). Milk urea nitrogen was reduced in PC relative to PO (*p* = 0.001).

Across periods, milk yield, ECM, and component yields were lower in Period 2 than in Period 1 (*p* < 0.05). Milk urea nitrogen was also greater in Period 1 (*p* = 0.001).

Milk fatty acid composition was not affected by supplementation, with no differences observed between PO and PC for total saturated (SFA; *p* = 0.38), monounsaturated (MUFAs; *p* = 0.24), or polyunsaturated fatty acids (PUFAs; *p* = 0.33). Palmitic acid (C16:0) was the predominant fatty acid across treatments. Period influenced the milk fatty acid profile, with lower SFA (*p* = 0.011) and PUFA (*p* = 0.003) and greater MUFA (*p* = 0.001) concentrations in Period 2. Omega-6 fatty acids tended to be higher under PO (*p* = 0.080), whereas omega-3 fatty acids were affected by period (*p* = 0.004) but not by treatment (*p* = 0.27).

### 3.5. Fecal, Urinary, Blood and Saliva pH and Fecal Particle Size

**Figure 2:**
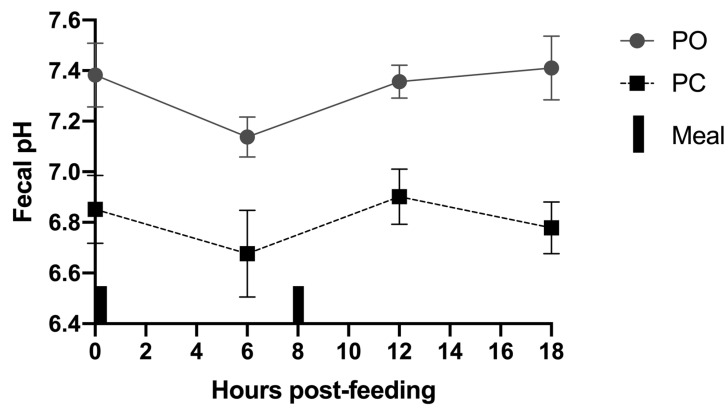
Diurnal pattern of fecal pH in cows fed pasture only (PO) or pasture plus concentrate (PC). Fecal pH was measured at 0, 6, 12, and 18 h relative to morning feeding. Values represent least squares means ± SEM. No treatment × sampling time interaction was detected (*p* > 0.05).

A diet × period interaction was detected for fecal pH (*p* = 0.02; Table 6): fecal pH was lower in PC than in PO in both periods, with a greater difference between treatments observed in Period 1 than in Period 2. The diurnal pattern of fecal pH differed between diets (Figure 2). No diet × period interactions were detected for urinary, blood, or salivary pH (*p* > 0.05), although a tendency for a period effect was observed for urinary pH (*p* = 0.063).

Regarding fecal particle size, a tendency for a diet × period interaction was observed for the proportion of particles > 4.75 mm (*p* = 0.09), with a greater proportion of particles in PC than PO during Period 1, whereas the opposite tendency occurred during Period 2. The proportion of intermediate particles (3.35–4.75 mm) was higher under PO than under PC (*p* < 0.05), whereas the proportion of particles < 1.4 mm tended to be greater under PC (*p* = 0.06). Across periods, the proportion of particles < 1.4 mm was greater in Period 2 than in Period 1 (*p* = 0.001).

### 3.6. Clinical Indicators

The effects of concentrate supplementation on clinical parameters are presented in Table 7. A treatment × period interaction was detected for respiration rate (*p* = 0.002), with higher values observed under PC than PO during Period 1, whereas no differences were detected in Period 2.

NEFA concentrations were lower under PC compared with PO (*p* = 0.023). Heart rate, rectal temperature, rumen motility, body weight, body condition score, fecal consistency, or glucose concentration did not differ significantly between treatments (*p* > 0.05). Across periods, heart rate was greater in Period 2 than in Period 1 (*p* = 0.039).

### 3.7. Enteric Methane Emissions

No treatment × period interactions were detected for methane emission parameters (*p* > 0.05; Table 8). Concentrate supplementation did not affect absolute methane production (g/d; *p* = 0.96). However, methane yield, expressed relative to dry matter intake (DMI), was lower under PC than PO (*p* = 0.002). Methane intensity was not affected by treatment (*p* = 0.24).

Across periods, absolute methane emissions increased in Period 2 compared with Period 1 (*p* = 0.005). Similarly, methane yield (g/kg DMI) was greater in Period 2 (*p* = 0.002), while methane intensity was also higher (*p* = 0.002).

## 4. Discussion

### 4.1. Dietary and Seasonal Effects on Ruminal Fermentation

Ruminal pH was influenced by both dietary treatment and experimental period. Concentrate supplementation was associated with a reduction in average ruminal pH; however, this response varied between periods, likely reflecting differences in pasture composition. A more pronounced reduction in minimum pH was observed in Period 1. Although, minimum ruminal pH did not fall below the commonly proposed SARA threshold, the discrete sampling approach based on measurements at 2 h intervals, may have underestimated short-term pH depressions occurring between sampling points; therefore, the absence of SARA should be interpreted considering this methodological limitation [6,28]. Continuous intraruminal pH monitoring would be required to confidently rule out episodic SARA.

A significant treatment × period interaction was detected for minimum ruminal pH (Table 4), indicating that the effect of concentrate supplementation was not uniform across experimental periods. During Period 1, when pasture quality was superior (Table 2), minimum ruminal pH was similar between PO and PC cows, suggesting that the buffering capacity associated with a highly digestible forage was sufficient to offset the additional fermentable substrate provided by the concentrate. In contrast, during Period 2, when pasture had matured and its nutritive value had declined substantially, minimum pH was lower in PC than in PO cows, indicating a more pronounced postprandial acidification under the combination of lower forage quality and concentrate supplementation. These results are consistent with previous reports indicating that the ruminal response to concentrate supplementation in pasture-based systems is strongly modulated by prevailing forage quality [13].

The reduction in ruminal pH with concentrate supplementation was accompanied by an increase in total VFA concentration [6,8]. Although specific carbohydrate fractions were not determined, this response is consistent with a greater supply of fermentable substrates associated with concentrate inclusion, which we infer from the ingredient composition of the concentrate rather than from direct chemical analysis of the diets. Nevertheless, minimum ruminal pH remained within the low 6 range across treatments. This suggests that ruminal buffering capacity and rumination activity were likely sufficient to maintain ruminal stability under the level of concentrate supplementation used in this study. Differences in the VFA profile likely reflect both dietary treatment and period-related variation in forage characteristics, which are known to influence ruminal fermentation [29,30]. Moorby et al. [29] reported linear increases in total VFA and butyrate concentrations and a decrease in acetate with increasing proportion of concentrate in dietary DM, while propionate molar percentage was not affected. In agreement with their findings, in the present study, propionate levels did not differ between treatments, whereas acetate was greater in cows receiving only pasture. The higher acetate molar percentage in the PO group reflects the predominance of cellulolytic fermentation pathways associated with high-forage diets [15].

Across periods, average ruminal pH was greater in Period 2 than in Period 1, accompanied by lower total VFA concentrations, reduced propionate, and a higher acetate proportion. This pattern is consistent with the lower digestibility and greater structural carbohydrate content of the more mature pasture offered later in the spring [31]. The reduction in aNDF digestibility supports the interpretation that forage maturation altered substrate availability for ruminal fermentation. The marked differences in pasture composition between periods indicate that pasture maturity was a major driver of the observed responses and likely interacted with the effect of concentrate supplementation; treatment effects are therefore interpreted against this seasonal background [30,32].

Ruminal ammonium concentrations were lower in Period 2 than in Period 1, likely reflecting the seasonal decline in pasture crude protein concentration and reduced ruminal proteolysis [33,34] and possibly also with improved synchronization between energy and nitrogen availability as pasture maturity advanced [35].

### 4.2. Digestibility and Nutrient Composition of Pastoral Diets

The chemical composition of pasture changed markedly between experimental periods, with declining CP concentration and digestibility and increasing DM content as spring progressed. These changes are consistent with the seasonal maturation of temperate swards, characterized by increased structural carbohydrate deposition and reduced leaf-to-stem ratio [36]. Similar declines in ryegrass digestibility with advancing maturity have been widely reported [37,38,39]. Total DMI was greater in cows receiving PC although supplementation partially replaced pasture intake, as reflected by substitution rates of 0.18 and 0.40 in Periods 1 and 2, respectively. These values fall within the expected range for grazing dairy cows [13,14,37] and indicate a moderate displacement of herbage that resulted in a net increase in total nutrient supply. The higher substitution observed in Period 2 is consistent with the more mature pasture conditions during this stage of the experiment. 

Concentrate supplementation did not modify apparent total-tract digestibility of DM, OM, or CP, whereas NDF digestibility decreased. Because minimum ruminal pH remained above the threshold classically associated with impaired growth of ruminal fibrolytic bacteria [38,39] a direct pH-mediated inhibition of fiber fermentation is unlikely. Other mechanisms, which were not evaluated here, are more plausible candidates: a faster digesta passage rate and reduced ruminal retention time for fiber fermentation [40], shifts in the fibrolytic microbial community, or reduced fibrolytic activity not captured by pH alone.

Across periods, NDF intake increased in Period 2 due to greater forage maturity and structural carbohydrate content and was accompanied by a reduced aNDF digestibility coefficient. Despite this, total tract digestibility of OM remained stable between periods, suggesting adaptive responses in intake behavior, rumination activity, or microbial efficiency that compensated for the reduced intrinsic forage quality and greater amount of forage ingested [40]. Overall, concentrate supplementation increased total nutrient supply, and fiber digestion responded differently to treatment and to seasonal pasture maturation.

It should also be noted that pasture was offered using a cut-and-carry approach during the 7-day measurement phase, following a 21-day rotational grazing adaptation period under each dietary treatment. Forage was not subjected to additional processing beyond cutting, minimizing potential alterations in physical structure; however, the transition to individual housing may have introduced some differences in feeding behavior, particularly with respect to selective grazing, compared with grazing conditions.

### 4.3. Effects of Concentrate Supplementation on Milk Yield and Composition

Concentrate supplementation increased both milk and ECM yields, with responses exceeding 3.5 kg/d between treatments, consistent with previous findings in grazing systems where improved energy density increases milk output [41,42]. The greater energy and nutrient supply associated with concentrate inclusion may enhance microbial protein synthesis and increase the availability of metabolizable nutrients to the mammary gland [43]. Milk production response to concentrate supplementation declined from 0.82 to 0.71 kg milk/kg concentrate DM in Periods 1 and 2, respectively, reflecting the combined effects of advancing lactation stage and declining pasture quality during spring [41,44]. 

Protein and fat yields were greater in supplemented cows, consistent with higher energy intake. The propionate–gluconeogenesis–amino acid sparing pathway has been proposed as a mechanism by which increased concentrate feeding may enhance milk protein synthesis [43,45]; however, this interpretation is offered as a physiological framework and is not directly supported by measurements conducted in the present study. Although fat yield increased, milk fat concentration remained unchanged. The absence of milk fat depression is consistent with the ruminal fermentation profile observed in the present study. The maintenance of adequate acetate and butyrate production, key precursors for *de novo* fatty acid synthesis [46] suggests that rumen conditions and effective fiber supply were sufficient to sustain mammary lipogenesis with the level of concentrate inclusion considered for this study [47,48,49]. Milk urea nitrogen concentrations were lower in supplemented cows and overall lower in Period 2, indicating improved energy–protein synchrony as lactation progressed and nutrient supply became more balanced [49,50]. 

Milk fatty acid composition was largely unaffected by concentrate supplementation, with no significant differences detected in major FA classes (SFA, MUFAs, PUFAs), consistent with previous studies using low to moderate levels of grain-based supplementation in grazing dairy cows [51,52]. However, PO cows exhibited a slightly higher omega-6 content, which may be attributed to a greater herbage intake rich in unsaturated fatty acids. In addition, seasonal variation influenced the milk FA profile, with decreased proportions of SFA and PUFA but increased MUFA in Period 2, potentially reflecting changes in pasture botanical composition and maturity [53,54]. The obtained NIRS estimates mainly represent major fatty acid fractions and do not capture odd- and branched-chain FA or specific biohydrogenation intermediates, such as trans-10 C18:1 and trans-10, cis-12 CLA [55]; therefore, subtle microbial-related changes in milk FA associated with the observed changes in ruminal fermentation cannot be excluded. 

### 4.4. Clinical and Metabolic Indicators 

Fecal pH has been proposed as an indicator of post-ruminal fermentation, but its interpretation is limited: it provides only indirect information on hindgut conditions, may also reflect differences in diet composition or digesta passage rate, and responds to ruminal events with a temporal delay [56]. In addition, most evidence for its use comes from studies under controlled feeding [57,58], with limited information available under grazing conditions. In this context, the lower fecal pH observed in concentrate-supplemented cows, together with a greater proportion of fine particles in their feces, is compatible with altered post-ruminal digestion, although the underlying mechanisms were not directly assessed here.

Urinary and salivary pH remained within physiological ranges and were not affected by treatment. These variables were included as descriptive indicators of systemic buffering only; they are not specific markers of ruminal acid–base status and should not be used to infer ruminal conditions [57,58].

Plasma NEFA concentrations were lower in supplemented cows, consistent with the greater energy intake associated with concentrate supplementation. Both treatment means remained below 300 µEq/L, within the reference range expected for cows in early to mid-lactation [59]. 

The lack of statistically significant differences in BW and BCS suggests that the experiment duration was insufficient for measurable changes in body reserves, despite metabolic improvements. Other clinical parameters, including rectal temperature, heart rate, rumen motility, and fecal consistency, remained within normal physiological ranges. Although a treatment × period interaction was detected for respiration rate, values remained within the physiological range reported for dairy cows [60,61], suggesting that the observed differences are unlikely to reflect clinically relevant alterations. 

### 4.5. Dietary and Seasonal Influences on Enteric Methane Emissions

Methane emissions were evaluated as a secondary outcome in the present study and should be interpreted within the methodological limitations of the experimental approach. Although the SF_6_ tracer technique is generally not recommended for rumen-fistulated cows due to potential alterations in eructation dynamics and rumen gas release through the cannula [62,63], fistulated animals were used here to allow periodic rumen pH sampling. Methane estimates should therefore be interpreted as indicative rather than quantitative, and results should not be directly compared with datasets obtained in non-fistulated animals.

Concentrate supplementation did not detectably affect daily methane output, in line with previous reports in pasture-based systems where supplementation increases intake without necessarily reducing daily methane output, because total fermentable substrate supply to the rumen remains comparable or increases [64,65,66]. Methane yield per unit of DM intake appeared lower in PC, consistent with the greater DMI in supplemented cows, and methane intensity did not differ between treatments, matching previous reports in grazing systems at comparable supplementation levels [44,65]. These between-treatment patterns are more robust than the absolute emission values, but they remain subject to the methodological caveats noted above and should be regarded as suggestive rather than conclusive.

Period effects on methane emissions were more pronounced than treatment effects. Methane output, yield, and intensity were all higher in Period 2, coinciding with lower pasture digestibility and greater fiber intake, conditions known to favor acetate production and hydrogen availability for methanogenesis [32,67]. The higher ruminal pH and fermentation pattern in Period 2 are consistent with a more fibrolytic rumen environment, which typically enhances methane formation [68,69]. Seasonal changes in pasture characteristics therefore had a greater impact on methane emissions than concentrate supplementation under the conditions of this study.

### 4.6. Limitations

It should be acknowledged that the relatively small sample size (*n* = 8) may limit statistical power and the generalizability of the results; however, the crossover design allowed each cow to serve as its own control, and *n* = 8 is typical for studies using rumen-cannulated cows [70,71].

## 5. Conclusions

Concentrate supplementation of dairy cows fed pasture-based diets modified the daily ruminal pH pattern, resulting in a moderate reduction in pH and increased fermentative activity, while minimum pH values remained within physiological limits. Under the conditions of the present study, based on discrete ruminal pH measurements, no evidence of subacute ruminal acidosis was detected indicating that the initial hypothesis was not supported. These responses were accompanied by the expected increase in dry matter intake associated with concentrate supplementation, as well as improvements in nutrient utilization, and milk yield, without adverse effects on clinical or physiological indicators. However, period effects on milk yield, ruminal pH, and VFA concentration were of comparable or greater magnitude than treatment effects, underscoring that seasonal variation in pasture characteristics was a primary determinant of the observed responses. Overall, the results suggest that concentrate supplementation may improve productive performance under the conditions of this study, but responses were strongly influenced by pasture maturity.

## Figures and Tables

**Table 1:** Ingredients and chemical composition of the experimental concentrate.

Ingredients Composition (g/kg of DM)	
Corn	450
Wheat bran	240
Whole oatmeal	140
Chickpea bran	100
Vinasse	30
Minerals ^1^	40
Chemical composition	
Dry matter (DM), g/kg	884.3
Crude protein, g/kg DM	141.9
Ash, g/kg DM	60.6
In vitro digestibility, g/kg DM	907.7
Metabolizable energy, MJ/kg DM	12.7

^1^ Nutrialmix containing per kg: Ca 180 g, P 50 g, Mg 60 g, Na 12 g, 1 g Se-enriched yeast, Cu 1200 mg, Mn 1500 mg, Zn 2700 mg, I 125 mg, Co 20 mg, and Se 30 mg.

**Table 2:** Chemical composition of pasture offered in the two periods of the experiment.

	Period 1	Period 2	SEM	*p*-Value
Dry matter (DM), g/kg	141.3	232.6	8.58	<0.001
Crude protein, g/kg DM	230.2	179.6	10.4	0.018
aNeutral detergent fiber ^1^, g/kg DM	464.4	467.3	11.8	0.870
Ash, g/kg DM	124.8	112.2	2.11	0.008
In vitro digestibility, g/kg DM	856.0	700.3	8.26	<0.001
Metabolizable energy, ^2^ MJ/kg DM	11.3	9.6	0.10	<0.001

^1^ Amylase-treated neutral detergent fiber; ^2^ metabolizable energy (ME) was estimated from in vitro digestible organic matter using the regression equation of [27]. SEM = standard error of the mean.

**Table 3:** Nutrient intake, apparent digestibility and digestible nutrient intake of dairy cows fed pasture only (PO) or pasture with concentrate (PC) diets.

	Period 1		Period 2			*p*-Value		
	PO	PC	PO	PC	SEM	Trt	Period	T × P
Nutrient intake, kg/d								
Pasture DM	12.7	11.8	15.8	13.7	0.87	0.08	0.01	0.42
Total DM	12.7	16.9	15.8	18.6	0.90	0.002	0.02	0.45
OM	11.1	14.6	14.2	16.7	0.78	0.003	0.006	0.52
CP	2.92	3.39	2.89	3.10	0.19	0.10	0.40	0.51
aNDF	5.91	5.47	7.51	6.40	0.40	0.08	0.008	0.43
Digestibility coefficient								
DM	0.77	0.79	0.73	0.78	0.019	0.18	0.22	0.45
OM	0.80	0.80	0.77	0.80	0.014	0.31	0.14	0.31
CP	0.80	0.77	0.79	0.78	0.019	0.29	0.95	0.49
aNDF	0.81	0.68	0.70	0.63	0.020	0.004	0.003	0.13

PO = pasture only; PC = pasture supplemented with concentrate; SEM = standard error of the mean; Trt = treatment; T × P = treatment × period interaction; DM = dry matter; OM = organic matter; CP = crude protein; aNDF = Amylase-treated neutral detergent fiber.

**Table 4:** The effect of concentrate supplementation on ruminal pH and volatile fatty acids (VFAs) of dairy cows.

	Period 1		Period 2			*p*-Value		
	PO	PC	PO	PC	SEM	Trt	Period	T × P
Average ruminal pH	6.41	6.42	6.87	6.58	0.06	0.032	0.007	0.20
Minimum ruminal pH	5.86 ^b^	5.87 ^b^	6.54 ^a^	6.07 ^b^	0.06	0.033	0.001	0.027
Maximum ruminal pH	7.01	7.22	7.31	7.12	0.09	0.87	0.13	0.31
Total VFAs, mM	116	121	66.8	78.2	3.09	0.005	<0.001	0.58
Acetate, mol/100 mol	71.7	69.5	73.6	70.7	0.54	0.001	0.021	0.71
Propionate, mol/100 mol	20.9	21.0	14.8	16.3	0.59	0.14	<0.001	0.53
Butyrate, mol/100 mol	6.68	8.77	9.68	11.01	0.37	0.001	<0.001	0.61
Valerate, mol/100 mol	0.56	0.66	0.78	0.87	0.03	0.035	0.001	0.88
Isobutyrate, mol/100 mol	0.38	0.32	0.60	0.57	0.01	0.039	<0.001	0.49
A:P ratio	4.91	4.60	5.12	4.48	0.40	0.29	0.92	0.77
NH_4_^+^ (mM)	10.9	9.27	5.72	6.11	0.95	0.14	<0.001	0.45

PO = pasture only; PC = pasture supplemented with concentrate; SEM = standard error of the mean; Trt = treatment; T × P = treatment × period interaction; A:P ratio = acetate to propionate ratio. Means within a row with different superscript letters differ (*p* < 0.05).

**Table 5:** Effect of concentrate supplementation on milk production and milk composition of dairy cows.

	Period 1		Period 2			*p*-Value		
	PO	PC	PO	PC	SEM	Trt	Period	T × P
Milk yield, kg/d	24.0	28.2	21.9	25.4	1.24	0.004	0.055	0.75
ECM yield, kg/d	26.3	30.1	22.6	27.7	1.17	0.001	0.013	0.58
Protein yield, kg/d	0.78	0.94	0.68	0.87	0.03	0.001	0.008	0.69
Fat yield, kg/d	0.99	1.09	0.81	1.02	0.05	0.002	0.010	0.25
Milk composition								
Protein, g/kg	32.5	33.8	31.1	34.4	0.62	0.001	0.54	0.12
Fat, g/kg	41.8	38.8	37.8	40.3	1.69	0.89	0.45	0.11
MUN, mg/100 mL	446	377	299	245	11.5	0.001	0.001	0.51
Fat to protein ratio	1.28	1.15	1.21	1.17	0.04	0.041	0.52	0.27
Fatty acid, g/100 g of total FA								
Ʃ SFA	72.1	73.1	69.2	70.0	1.01	0.38	0.011	0.95
C8:0	0.71	0.62	0.72	0.77	0.01	0.15	0.68	0.22
C10:0	3.44	3.08	2.37	2.82	0.18	0.79	0.003	0.042
C12:0	4.59	4.34	3.43	3.84	0.24	0.73	0.004	0.19
C14:0	14.5	15.2	13.1	13.7	0.81	0.58	0.069	0.82
C16:0	31.7	30.6	31.9	33.1	0.80	0.99	0.11	0.17
C18:0	10.3	9.3	9.5	10.7	0.53	0.91	0.60	0.064
Ʃ MUFA	24.3	23.6	28.8	27.1	0.97	0.24	0.001	0.61
c-9-18:1	12.9	10.3	18.2	18.8	0.79	0.22	0.001	0.07
c-9 t-11-18:2	0.44	0.47	1.27	1.06	0.07	0.22	0.001	0.096
Ʃ PUFA	3.62	3.33	2.01	2.87	0.28	0.33	0.003	0.06
*Ʃ n − 6*	1.39	1.20	1.41	1.36	0.06	0.080	0.16	0.25
*Ʃ n − 3*	0.71	0.68	0.84	0.79	0.03	0.27	0.004	0.72
*20:5 n − 3*	0.06	0.06	0.08	0.08	0.005	0.58	0.001	0.87
*22:5 n − 3*	0.08	0.08	0.10	0.09	0.004	0.37	0.006	0.62

PO = pasture only; PC = pasture supplemented with concentrate; SEM = standard error of the mean; Trt = treatment; T × P = interaction treatment × period; ECM = Energy corrected milk yield = (0.327 × Milk Yield) + (12.69 × Milk Fat Yield) + (7.65 × Milk Protein Yield). MUN = milk urea nitrogen; SFA = saturated fatty acids; MUFAs = monounsaturated fatty acids; PUFAs = polyunsaturated fatty acids.

**Table 6:** Effect of concentrate supplementation on fecal, urinary, blood and saliva pH of dairy cows.

	Period 1		Period 2			*p*-Value		
	PO	PC	PO	PC	SEM	Trt	Period	T × P
pH								
Fecal	7.15 ^b^	6.50 ^c^	7.49 ^a^	7.10 ^b^	0.04	0.001	0.001	0.02
Urinary	8.25	8.29	8.42	8.43	0.08	0.78	0.063	0.85
Blood	7.62	7.58	7.57	7.58	0.02	0.55	0.39	0.47
Saliva	8.55	8.55	8.72	8.51	0.06	0.22	0.42	0.19
Particle fraction (%) fecal								
>4.75 mm	18.9	25.1	15.6	10.9	3.21	0.83	0.009	0.09
3.35–4.75 mm	21.7	15.9	17.9	14.2	2.16	0.035	0.21	0.63
<1.4 mm	59.4	59.0	66.5	74.9	3.14	0.06	0.001	0.16

PO = pasture only; PC = pasture supplemented with concentrate; SEM = standard error of the mean; Trt = treatment; T × P = interaction treatment × period. Means within a row with different superscript letters differ (*p* < 0.05).

**Table 7:** Effect of concentrate supplementation on clinical parameters of dairy cows.

	Period 1		Period 2			*p*-Value		
	PO	PC	PO	PC	SEM	Trt	Period	T × P
Heart rate, beats/min	58.6	68.2	69.1	69.6	2.84	0.080	0.039	0.11
Respiration rate, breaths/min	22.1 ^b^	30.0 ^a^	29.1 ^a^	29.1 ^a^	1.85	0.019	0.039	0.002
Rectal temperature, °C	38.1	38.3	38.1	38.1	0.009	0.34	0.30	0.24
Rumen motility, contractions per min	2.75	2.5	2.65	2.75	0.30	0.84	0.84	0.54
Average body weight, kg	520	504	553	538	27.3	0.25	0.58	0.98
BCS	2.7	3.03	2.88	2.72	0.11	0.51	0.76	0.06
Fecal consistency	1.5	1.5	1.75	1.5	0.22	0.57	0.57	0.57
Glucose, mg/dL	54.7	56.1	55.6	54.0	3.10	0.96	0.85	0.64
NEFA, µEq/L	224	107	198	90	50.8	0.002	0.29	0.85

PO = pasture only; PC = pasture supplemented with concentrate; SEM = standard error of the mean; Trt = treatment; T × P = interaction treatment × period; BCS = body condition score was on a scale of 1 to 5 (1 = emaciated and 5 = extremely fat, using 0.25 increments). Means within a row with different superscript letters differ (*p* < 0.05).

**Table 8:** Effect of concentrate supplementation on methane emissions of dairy cows.

	Period 1		Period 2			*p*-Value		
	PO	PC	PO	PC	SEM	Trt	Period	T × P
CH_4_ outputs (g/d)	204	223	314	294	16	0.96	0.005	0.46
CH_4_/DMI (g/kg)	15.5	13.0	20.5	15.7	0.6	0.002	0.002	0.36
CH_4_/milk yield (g/kg)	7.58	8.31	14.2	11.4	0.8	0.24	0.002	0.25

PO = pasture only; PC = pasture supplemented with concentrate; SEM = standard error of the mean; Trt = treatment; T × P = interaction treatment × period; DMI: dry matter intake.

## Data Availability

All data obtained in this study are available in the article.

## Abbreviations

The following abbreviations are used in this manuscript:

SARA	Subacute ruminal acidosis
VFAs	Volatile fatty acids
NEFA	Non-esterified fatty acids
PO	Pasture only
PC	Pasture plus concentrate
DM	Dry matter
CP	Crude Protein
aNDF	Amylase-treated neutral detergent fiber
OM	Organic matter
ECM	Energy-corrected milk
DMI	Dry matter intake
BW	Body weight
BCS	Body condition score
MUN	Milk urea nitrogen
SFAs	Saturated fatty acids
MUFAs	Monounsaturated fatty acids
PUFAs	Polyunsaturated fatty acids
